# Comparison of Different Scoring Systems in Predicting the Severity of Acute Pancreatitis: A Prospective Observational Study

**DOI:** 10.7759/cureus.6943

**Published:** 2020-02-10

**Authors:** N. R. Venkatesh, Chellappa Vijayakumar, Gopal Balasubramaniyan, Sakthivel Chinnakkulam Kandhasamy, Sudharsanan Sundaramurthi, Sreenath G. S., Krishnamachari Srinivasan

**Affiliations:** 1 Surgery, Jawaharlal Institute of Postgraduate Medical Education and Research, Puducherry, IND

**Keywords:** modified glasgow score, acute pancreatitis, apache ii score, bisap score, ranson score, procalcitonin

## Abstract

Background

Acute pancreatitis (AP) is an inflammatory condition of the pancreas mostly due to alcohol or gallstones. Various scoring systems were involved in identifying the severity of the disease. The standard single score to identifying the severity remains uncertain.

Methodology

This prospective observational study was carried out for two years in a tertiary care center from South India. The diagnosis of AP was made based on Atlanta criteria, and a total of 164 patients were included. All patients were assessed by acute physiology and chronic health evaluation ll (APACHE II), bedside index for severity in AP (BISAP), modified Glasgow score (MGS), and Ranson score on admission and 48 hours after admission scores. Procalcitonin was done in all patients with AP. Contrast-enhanced computed tomography (CT) of the abdomen was done in 69 patients who had features of severe acute pancreatitis (SAP). Sensitivity, specificity, positive predictive value (PPV), negative predictive value (NPV), and diagnostic accuracy were calculated for each score, and procalcitonin for CT documented severe patients and organ failure patients together.

Results

A total of 164 patients were included in this study. CT abdomen showed a modified CT severity index (MCSI) ≥8 in all 69 (100%) patients. APACHE II score could predict SAP based on CT findings in 44 patients (63.76%), BISAP score in 22 patients (31.88%), MGS in 55 patients (79.71%), Ranson score at admission in 31 patients (44.92%), Ranson score 48 hours after admission in 44 patients (63.76%), and procalcitonin on admission in 69 patients (100%) when cut-off used as per the literature. APACHE II score could predict SAP in cases of AP (n=164) in 52 patients (50%), BISAP score in 27 patients (26%), MGS in 79 patients (76%), Ranson score at admission in 34 patients (33%), and Ranson score 48 hours after admission in 61 (59%) patients when cut-off was used as per the literature. This study demonstrated that Ranson score on admission had a good area under the curve (AUC). AUC (0.8483), APACHE II (AUC 0.7708), and Ranson score 48 hours after admission (AUC 0.8167) had a fair accuracy. BISAP (AUC 0.6399) and MGS (AUC 0.6486) had poor accuracy for the prediction of severity in AP based on receiver operator characteristic (ROC) curves.

Conclusion

Among the scoring system compared, MGS had the highest sensitivity for predicting the severity of AP. However, Ranson score on admission had better diagnostic accuracy for predicting severity, organ failure, and mortality based on ROC curves. Procalcitonin had the best sensitivity, specificity, PPV, NPV, and diagnostic accuracy for association with severity in AP.

## Introduction

Acute pancreatitis (AP) is a sudden inflammation of the pancreas, which is characterized by the activation of pancreatic enzymes to cause self-digestion of the pancreas. It is an acute inflammatory process presenting as a mild discomfort with local inflammation to severe disease with multi-organ failure. It has a mortality of approximately 1% among all AP but so high as 20% to 30% among those with severe acute pancreatitis (SAP), which is a process of acute inflammation of the pancreas with the involvement of regional tissues or organ systems [1]. AP is a common clinical condition, yet no prevalence data are not available from India. Only incidence is available from tertiary centers 55 patients per year [1]. The incidence of AP has been reported to be higher in the USA, Finland, and Scotland (49.3, 46.6, and 41.9 per 100,000 population, respectively) [2].

Gallstones and alcohol are the most common causes of AP in India [3]. Other causes are hypercalcemia, drug-induced pancreatitis, and dyslipidemia. Smoking also has been found to be a cause in 30% of the patients which also carries higher mortality (20%) [4]. A study from Sweden invited for a health questionnaire, which found smoking was associated with AP with a relative risk of 3.57 among those who had no history of alcohol consumption [5]. There are several indices in use to evaluate pancreatitis patients. Therefore, an attempt has been made to identify which scoring system predicts the severity in AP in this study. Secondarily, it aimed to assess the correlation between procalcitonin level and severity of AP.

## Materials and methods

This prospective observational study was carried out for two years in a tertiary care center from South India. Patients who presented with acute abdomen were examined, and in suspected cases of pancreatitis, serum amylase along with ultrasonography of the abdomen was done. The diagnosis of AP was made based on the Atlanta criteria, and a total of 164 patients were included. Informed consent was obtained from all participants, and this study was approved by the Institute Ethics Committee. All patients with chronic pancreatitis and those who were treated outside before presenting to the emergency were excluded from the study.

Various clinical and biochemical parameters were studied on admission and 48 hours after admission. Data were collected regarding demographics, detailed history, and physical examination, including complete hemogram, liver function test, and procalcitonin levels. Procalcitonin value of 0.5 ng/mL was accounted as the cut-off value for identifying the severity of AP as per the literature.

Patients were managed as per the standard institute guidelines. Patients who improved within 72 hours were labeled mild AP. If symptoms persisted after 72 hours, or no clinical improvement was there, contrast-enhanced computed tomography (CT) of the abdomen was done for those without organ failure. CT findings were graded as per the modified CT severity index (MCSI). CT findings and/or evidence of organ failure were taken as the gold standard for diagnosing severity using the Atlanta criteria, and it was used to compare four scores. All patients were assessed for acute physiology and chronic health evaluation (APACHE) II score, bedside index for severity in AP (BISAP), modified Glasgow score (MGS), and Ranson score on the first 24 hours and 48 hours after to it. Patients were followed up until discharge or death.

Statistical analysis

OpenEpi statistical software was used to analyze the data, and the receiver operator characteristic (ROC) curve was plotted with all the scores and procalcitonin using the data generated. Sensitivity, specificity, positive predictive value (PPV), negative predictive value (NPV), and diagnostic accuracy of all four scores for predicting CT diagnosed severity, organ failure, clinical severity, and mortality were compared.

## Results

A total of 224 patients with upper abdominal pain and referred to casualty were investigated. Among them, 164 patients had features of AP, according to the Atlanta classification. Of these 164 patients, 60 (36.58%) were diagnosed as mild AP, 104 (63.41%) were diagnosed as having SAP, 35 (33.65%) developed organ failure before 72 hours, and 69 (66.3%) underwent CT abdomen after 72 hours (based on the Atlanta classification severity). A total of 63 patients (60.57%) needed intensive care admission, 15 (12.5%) died during hospitalization, and four went against medical advice. The mean age of patients at presentation was 45.09 years (range 15-85). 

Etiology of pancreatitis 

Alcohol was found to be a significant cause of AP, and it was found in 115 (70.1%) patients (Table 1).

**Table 1:** Etiology of pancreatitis in study patients

Etiology	No of patients (n=164)
Alcohol	115 (70.1%)
Gallstone disease	33 (20.12%)
Idiopathic	15 (9.1%)
Hypertriglyceridemia and gallstone disease	20 (12.2%)
Trauma	1 (0.06%)

Modified CT severity index

CT abdomen in 69 patients showed MCSI ≥8 in all 69 (100%) patients (Table 2).

**Table 2:** Complications diagnosed with gold standard CT abdomen in study patients MCSI: modified CT severity index

Complications	No of patients (n=69)
Acute fluid collection	56 (81.15%)
Necrotizing pancreatitis	49 (71.01%)
Splenic vein thrombosis	12 (17.39%)
Pleural effusion/ascites/gastrointestinal involvement	60 (86.95%)
Portal vein thrombosis	5 (7.2%)
Distal superior mesenteric vein thrombosis	1 (1.44%)
MCSI ≥8	69 (100%)

Comparison of scoring systems for prediction of severity in CT documented SAP patients

Among the scoring systems, MGS had the highest sensitivity to predict severity as per CT findings. Ranson score at admission had the highest specificity and PPV. APACHE II and MGS had the highest diagnostic accuracy. Procalcitonin had the highest sensitivity, specificity, PPV, and diagnostic accuracy for CT documented severity (Table 3).

**Table 3:** Comparing sensitivity, specificity, PPV, NPV, and diagnostic accuracy for four scores for 69 CT severity cases based on literature cut-off values PPV: positive predictive value; NPV: negative predictive value; APACHE: acute physiology and chronic health evaluation; BISAP: bedside index for the severity in acute pancreatitis; MGS: modified Glasgow score; CI: confidence interval

Scoring system	Sensitivity, % (95% CI)	Specificity, % (95% CI)	PPV, % (95% CI)	NPV, % (95% CI)	Diagnostic accuracy, % (95% CI)
APACHE II	63.7 (51.9-74.1)	77.1 (60.98-87.93)	84.6 (72.4-91.9)	51.9 (38.69-64.9)	68.2 (58.81-76.43)
BISAP	31.8 (22.09-43.58)	85.7 (70.62-93.74)	81.4 (63.3-91.82)	38.9 (28.84-50.13)	50 (40.56-59.44)
MGS	79.9 (68.78-87.51)	31.4 (18.55-47.98)	69.6 (58.77-78.66)	44 (26.67-62.93)	63.4 (53.88-72.08)
Ranson at admission	44.9 (33.77-56.62)	91.4 (77.62-97.04)	91.1 (77.04-96.95)	45.7 (34.57-57.3)	60.5 (50.97-69.43)
Ranson at 48 hours	63.7 (51.9-74.1)	51.4 (35.57-67.01)	72.1 (59.83-81.81)	41.8 (28.38-56.67)	59.6 (50.01-68.54)
Procalcitonin	89.6 (82.79-93.38)	100 (92.59-100)	100 (96.44-100)	80 (68.22-88.17)	92.6 (87.65-95.77)

Comparison of scoring systems in predicting SAP based on literature cut-off values

Among the scoring systems, MGS had the highest sensitivity, NPV, and diagnostic accuracy, and all the four scores had better specificity and PPV as per the literature cut-off values for predicting severity in 164 AP patients. Procalcitonin had the highest sensitivity, NPV, and diagnostic accuracy as per the literature cut-off values for predicting severity in 164 AP patients (Table 4).

**Table 4:** Diagnostic values of four scoring systems and procalcitonin when all acute pancreatitis patients compared PPV: positive predictive value; NPV: negative predictive value; APACHE: acute physiology and chronic health evaluation; BISAP: bedside index for the severity in acute pancreatitis; MGS: modified Glasgow score; CI: confidence interval

Scoring system	Sensitivity, % (95% CI)	Specificity, % (95% CI)	PPV, % (95% CI)	NPV, % (95% CI)	Diagnostic accuracy, % (95% CI)
APACHE II	50 (40.56-59.44)	100 (93.98-100)	100 (93.12-100)	53.57 (44.37-62.54)	68.29 (60.82-74.93)
BISAP	25.96 (18.5-35.14)	100 (93.98-100)	100 (87.54-100)	43.8 (35.77-52.16)	53.05 (45.43-60.53)
MGS	75.96 (66.92-83.15)	100 (95.36-100)	100 (95.36-100)	70.59 (60.18-79.21)	84.76 (78.46-89.46)
Ranson at admission	32.69 (24.43-42.18)	100 (93.98-100)	100 (89.85-100)	46.15 (37.82-54.71)	57.32 (49.66-64.63)
Ranson at 48 hours	58.65 (49.05-67.65)	100 (93.98-100)	100 (94.08-100)	58.25 (48.6-67.31)	73.78 (66.56-79.91)
Procalcitonin	89.66 (82.79-93.38)	100 (92.59-100)	100 (96.44-100)	80 (68.22-88.17)	92.68 (87.65-95.77)

 

ROC curves plotted using the data for four scores and procalcitonin

**Figure 1:**
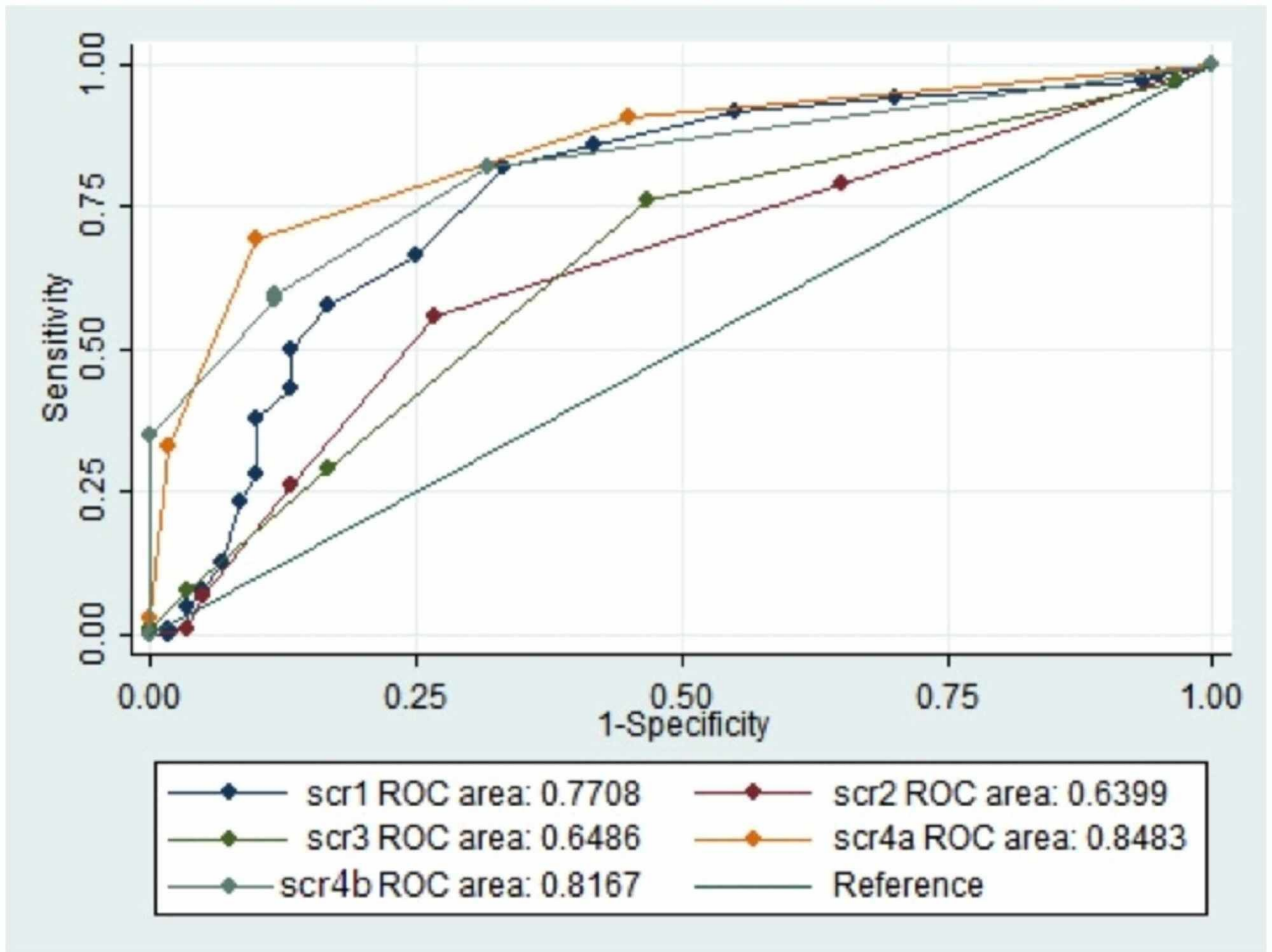
ROC curves four scores and procalcitonin in study patients ROC: receiver operator characteristic; score 1: acute physiology and chronic health evaluation II; score 2: bedside index for the severity in acute pancreatitis; score 3: modified Glasgow score; score 4a: Ranson score at admission; score 4b: Ranson score 48 hours after admission.

On the basis of the highest sensitivity and specificity values generated from the ROC curves, the following cut-offs were selected for further analysis: Ranson ≥2, Glasgow ≥3, BISAP ≥2, APACHE II ≥6, and procalcitonin ≥1.5 ng/mL. Ranson score on admission had the highest area under the curve (AUC) based on the ROC curve to predict SAP among the four scoring systems (Figure 1).

Comparison of scoring systems in SAP patients with cut-off points generated by ROC curves 

When a cut-off for APACHE II ≥8 was used as per the literature, it could predict severity in 69 (66.3%) patients, whereas when the cut-off was improved to ≥6 based on the ROC curve from this study, we could predict severity in 85 (81.7%) AP patients (Table 5).

**Table 5:** Comparison of scoring systems in SAP patients with cut-off points generated by ROC curves APACHE: acute physiology and chronic health evaluation; BISAP: bedside index for the severity in acute pancreatitis; MGS: modified Glasgow score; ROC:  receiver operator characteristic

Scoring system	With literature cut-off	With ROC curve generated study cut-off
APACHE	66.34% (n=69)	81.7% (n=85)
BISAP	21.15% (n=22)	55.76% (n=58)
MGS	52.58% (n=55)	52.58% (n=55)
Ranson at admission	29.80% (n=31)	75.96% (n=79)
Ranson at 48 hours	34.61% (n=44)	42.30% (n=62)
Procalcitonin	100% (n=104)	100% (n=104)

Comparison of scoring systems for association with organ failure 

APACHE II and MGS had the highest sensitivity and diagnostic accuracy in predicting organ dysfunction. Ranson score had the highest specificity. Procalcitonin had the highest specificity, sensitivity, PPV, NPV, and diagnostic accuracy for association with organ failure (Table 6).

**Table 6:** Comparison of scoring systems for association with organ failure in study patients APACHE: acute physiology and chronic health evaluation; BISAP: bedside index for the severity in acute pancreatitis; MGS: modified Glasgow score

Scoring system	Sensitivity, %	Specificity, %	PPV, %	NPV, %	Diagnostic accuracy, %
APACHE II	48.5	36.2	27.8	58.1	40.3
BISAP	8.5	55	8.8	54.2	39.4
MGS	68.5	20.2	30.3	56	36.5
Ranson at admission	14.2	68.1	18.5	61	50
Ranson at 48 hours	22.8	36.2	15.3	48	31.7
Procalcitonin	100	100	100	100	100

Mortality among AP patients predicted by various scores 

APACHE II score was associated with mortality in SAP in 12 (63.15%) patients when a cut-off ≥8 was used as per the literature, but when the cut-off was improved to ≥10 based on the ROC curve from this study, the association was in eight (42.1%) patients (Table 7).

**Table 7:** Mortality among acute pancreatitis patients predicted by various scores APACHE: acute physiology and chronic health evaluation; BISAP: bedside index for the severity in acute pancreatitis; MGS: modified Glasgow score; ROC: receiver operator characteristic

Scoring system	With literature cut-off	With ROC curve generated study cut-off
APACHE	63.15% (n=12)	42.1% (n=8)
BISAP	42.1% (n=8)	68.4% (n=13)
MGS	63.1% (n=12)	63.1% (n=12)
Ranson at admission	26.3% (n=5)	52.6% (n=10)
Ranson at 48 hours	52.63% (n=10)	57.89% (n=11)
Procalcitonin	15% (n=3)	47% (n=9)

During follow-up, scores and procalcitonin during the first admission in AP did not have much bearing in the prediction of chronicity.

## Discussion

AP is an inflammatory condition of the pancreas and may have a variable severity. Most of the patients have mild disease with minimal morbidity, and the rest of the patients have 10%-20% of mortality in SAP [6]. In this study based on MCSI, there were 69 (42%) SAP patients who are similar to Bezmarevic et al. study [7]. Cho et al. in their study of 161 AP patients reported that 52 patients with SAP had APACHE II score ≥8 similar to this study [8]. Khanna et al. reported higher sensitivity, specificity, and diagnostic accuracy for APACHE II score ≥8 for predicting severity [9]. Similar to this study, they reported that the APACHE II score had the best AUC for association with mortality [9].

Similar to this study, Cho et al. in their study reported that BISAP score ≥3 predicted SAP and increased mortality [8]. In their study, they reported that patients with BISAP score ≥3 had 76.1 more times a chance to develop SAP and 121.7 times associated with mortality [9]. Five SAP patients with organ failure had BISAP score ≥3, similar to this study [10]. Khanna et al. reported that BISAP scores ≥3 had higher sensitivity (74%) but less specificity (68%) than this study [9]. Park et al. concluded that the BISAP score of 2 was significant statistically for predicting SAP, organ failure, and mortality [11]. AUC for BISAP for predicting severity in AP was 0.8, and for mortality, it was 0.86 [11]. AUC for Ranson score predicting the severity of AP was 0.74 and for mortality 0.74 [11]. In contrast to this study, the BISAP score had better accuracy for SAP.

Similar to this study, Khanna et al. reported that the MGS had diagnostic accuracy was 75% for predicting SAP [9]. Khanna et al. also reported that the Ranson score had better AUC for predicting severity [9]. Papachristou et al. reported that Ranson score had better AUC for predicting severity (0.94) and mortality (0.95), in comparison to this study [12]. Cho et al. in their study found that AUC for Ranson score for predicting severity in AP was 0.804 (0.717-0.892) with a sensitivity of 81.8%, specificity of 59.1%, and PPV of 76.9% and for association with mortality 0.861 (0.734-0.988) with sensitivity of 87.5%, specificity of 57.2%, and PPV of 5.3% [8]. Three SAP patients with organ failure had Ranson score ≥3 on admission and 17 patients had Ranson score ≥3 after 48 hours of admission, in comparison to our study [10]. Simoes et al. reported that the Ranson score had a higher sensitivity of 91.2% in predicting severity, but had lesser specificity compared to this study [13]. Kim et al. reported that the Ranson score had the highest accuracy based on AUC [14]. Woo et al. reported that 3.29 ng/mL had better accuracy for predicting severity [15]. Khanna et al. reported that procalcitonin had an AUC of 0.88 for predicting severity [9]. 

The limitations of this study results were the use of the original Atlanta classification in place of the revised Atlanta classification, and procalcitonin was measured only once on the day of admission. Sensitivity and specificity were done using the Wilson method using OpenEpi online calculator.

## Conclusions

MGS had the highest sensitivity for predicting the severity of AP. However, Ranson score at admission had better diagnostic accuracy for predicting severity, organ failure, and mortality based on ROC curves. Procalcitonin had the best sensitivity, specificity, PPV, NPV, and diagnostic accuracy for association with severity in AP. BISAP score may be calculated within 24 hours of admission, but APACHE II and MGS had better diagnostic accuracy. Ranson score at admission is the best one for prediction of severity in AP among the four scores. APACHE II score is the best one for association with mortality in SAP patients. Procalcitonin on admission had the best sensitivity, specificity, PPV, and diagnostic accuracy for predicting severity in AP, organ failure, and mortality. 
